# Information assessment of hospital websites in Croatia: How to develop accountability standards?

**DOI:** 10.1002/hpm.2975

**Published:** 2020-03-30

**Authors:** Sanja Seljan, Maja Baretić, Marko Seljan, Mirjana Pejić Bach

**Affiliations:** ^1^ Faculty of Humanities and Social Sciences, Information and Communication Sciences University of Zagreb Zagreb Croatia; ^2^ Department of Endocrinology and Diabetology, School of Medicine, University Hospital Centre Zagreb University of Zagreb Zagreb Croatia; ^3^ Faculty of Economics and Business University of Zagreb Zagreb Croatia

Dear Editor

We have read with great interest the article from Asya Cooley highlighting that organizational website is the first and most trusted resource healthcare stakeholders turn to when in need of information so we would like to share our experiences regarding evaluation of hospital websites in Croatia.^1^

We evaluated websites from 28 state hospitals in Croatia. The analysis was performed using a Codebook including 89 items divided into five categories: *Technical characteristics*, *Hospital information and facilities*, *Medical services*, *Interactive on‐line services*, *and External activities*.^2^ The aim was to score each website by identifying the presence or absence of the 89 items (1 = item found, 0 = item not found). The website evaluation score was calculated on the basis of the items satisfied and compared two subgroups of hospitals ranked according to *Croatian* Institute of Public Health (Group 1 included seven national and six county hospitals of regional importance and Group 2 included 12 county and 3 local hospitals).^3^ Hospitals from Group 2 had significantly less beds then those form Group 1 (*P* = .001), though there was no difference in bed occupancy. The overall median website score was 33.5 (11‐48). There was no statistically significant difference in two subgroups regarding total hospital websites score, but statistical significance was present among two groups with regard to *Hospital information and facilities* (*P* = .038) and *External facilities* (*P* = .001).

Total evaluation score pointed to poor quality of hospital websites in Croatia. There were two distinctive categories of web quality: *Hospital information and facilities* (items concerning general information, history of the hospital, location, ways of reaching hospital, contact details of public relations office, International Organization for Standardization (ISO) certification, patients' rights and obligations, complementary services) and *External facilities* (items concerning ability to obtain health information, job opportunities, list of conferences, presence in media, publications, existence of courses, studies, booklets for download). It seems that the category *Hospital information and facilities* depends more on personal engagement of web‐master, but the category *External facilities* points that higher ranked hospitals (mostly academic ones) offer information beyond basic healthcare.

As stated in the article, we refer to Reference 1, well‐established online accountability practices will lead to public expectations on how a hospital should account for and justify its actions to various stakeholders. In Croatia, there is still no regulatory framework recommending accountability standards of hospital websites. We propose that standards for local and county hospitals website should provide concise and clear information and standards for highly specialized/academic hospitals should strive to provide also online accountability landscape.

## CONFLICT OF INTEREST

All authors declare no conflict of interest.
